# Improving a Tn7-based luciferase reporter system for promoter activity studies

**DOI:** 10.1099/mic.0.001655

**Published:** 2026-01-21

**Authors:** Brenno Wendler Miranda, Cristina Elisa Alvarez-Martinez

**Affiliations:** 1 Departamento de Genética, Evolução, Microbiologia e Imunologia, Instituto de Biologia, Universidade Estadual de Campinas (UNICAMP), Campinas, SP, Brazil

**Keywords:** *luxCDABE*, Tn7 vectors, type III secretion system (T3SS), *Xanthomonas citri*

## Abstract

Single-copy chromosomal integration systems are essential tools for stable gene expression in bacteria, minimizing variability associated with plasmid-based systems. The Tn7 transposon-based system is widely used for this purpose, and one important application is the generation of reporter systems, such as the bioluminescent *luxCDABE* operon (*lux*). However, current Tn7-lux vectors exhibit undesirable background expression due to cryptic promoter activity near the antibiotic resistance cassette. Here, we report the construction of an improved vector, pTn7-lux-B0015, incorporating a strong synthetic terminator upstream of the *lux* operon. This modification effectively eliminated basal luminescence in the absence of a promoter and enhanced the dynamic range and responsiveness of the reporter. Using a *Xanthomonas citri* type III secretion system promoter as a model, we demonstrate that pTn7-lux-B0015 enables more accurate detection of gene expression under relevant growth conditions. This vector provides a valuable tool for the development of precise and tunable bioluminescent reporters in bacterial systems.

## Introduction

Single-copy chromosomal gene expression systems are valuable tools for studying and engineering bacterial species because they reduce metabolic burden and minimize copy-number fluctuation effects on reporter output. A widely used approach for single-copy insertion is the Tn7 transposon-based chromosomal integration system, which inserts sequences located between transposase recognition sites into the conserved genomic site downstream of the *glmS* gene. Tn7-derived vectors have been adapted for multiple applications, including heterologous gene expression, stable strain labelling and creation of transcriptional and translational fusions [1].

**Fig. 1.**
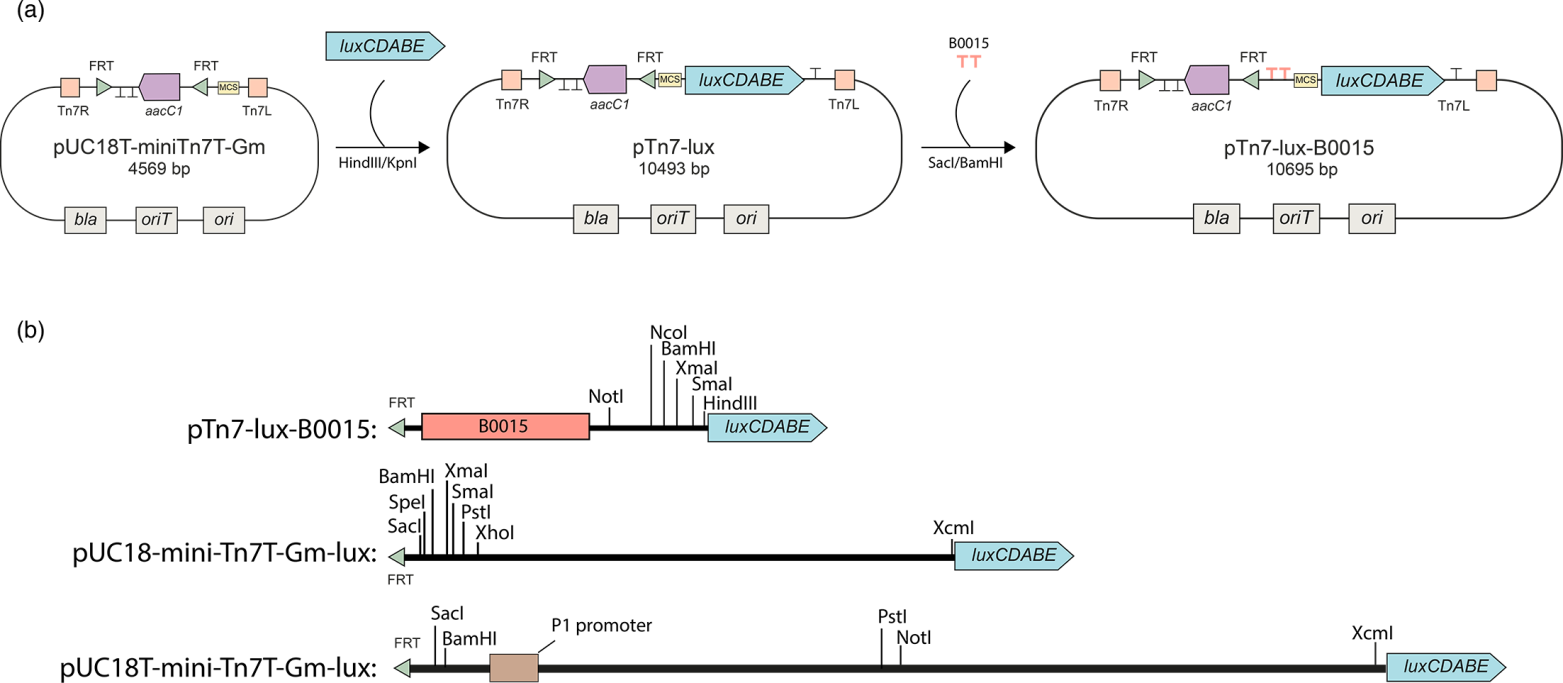
Tn7-lux plasmids. (**a**) Construction of pTn7-lux-B0015. First, the *luxCDABE* operon was cloned into pUC18T-mini-Tn7T-Gm, generating pTn7-lux. Then the double terminator B0015 was cloned between SacI and BamHI to create pTn7-lux-B0015. (**b**) Comparison of MCS regions, defined as the sequence between the FRT site upstream of the *aacC1* gene and the start codon of *luxC*. The pTn7-lux-B0015 contains a 297 bp MCS, of which 115 bp correspond to the double terminator B0015. pUC18-mini-Tn7T-Gm-lux [1] has 543 bp MCS and pUC18T-mini-Tn7T-Gm-lux [2] has a 970 bp MCS. MCSs are in scale, whereas FRT sites and the *luxCDABE* operon are not.

Among these constructs, pUC18-mini-Tn7T-Gm-lux and its conjugative derivative pUC18T-mini-Tn7T-Gm-lux were developed to generate transcriptional fusions to the *luxCDABE* bioluminescent operon; the latter places the reporter under a P1 promoter for constitutive expression in certain contexts [2]. Despite being widely utilized, these vectors possess features that can confound quantitative readouts obtained from bioluminescent genetic sensors. Notably, they display unexpectedly high luminescence in the absence of a cloned promoter (i.e. in promoterless insertions) [3] and include uncharacterized DNA segments (ranging from 543 to 970 bp) located between the flippase recombinase target (FRT) site and the *luxC* start codon (Fig. 1). Such cryptic regulatory elements compromise sensor sensitivity and dynamic range and may obscure bona fide promoter-driven responses. A prior study demonstrated that deletion of the sequence between the multiple cloning site (MCS) and the *luxCDABE* operon in pUC18-mini-Tn7T-Gm-lux reduced basal expression while increasing maximum inducible expression, consistent with the presence of an intrinsic promoter and promoter interference effects [4].

Here, we describe the rationale, design and validation of a redesigned Tn7-based lux reporter, pTn7-lux-B0015, with minimized uncharacterized sequence upstream of the *lux* operon and with a strong synthetic double terminator upstream of the MCS to block upstream transcriptional readthrough. We validated this construct using chromosomal integrations in *Xanthomonas citri* pv. *citri* (*X. citri*) and reporter fusions to the promoter of the type III secretion system (T3SS) structural gene *hrcU*, assessing its performance under conditions known to differentially regulate T3SS gene expression [5,6]. The results demonstrate that insertion of a robust terminator upstream of the reporter abolishes cryptic promoter activity, substantially improves signal specificity and enhances dynamic range and temporal responsiveness to carbon source changes. Because insertion of the terminator blocks cryptic readthrough from sequences adjacent to the antibiotic resistance cassette and FRT sites, flippase-mediated excision of the resistance marker is not required to effectively use this plasmid.

## Methods

### Plasmid construction

The *luxCDABE* operon was PCR-amplified from pGLR1 [7] using primers *pGLR-lux-fwd* and *pGLR-lux-kpni-rev*, digested with HindIII and KpnI and ligated into pUC18T-mini-Tn7T-Gm [1] to generate pTn*7-lux*. The double terminator B0015 was PCR-amplified from the pSB1C3-B0015 plasmid (BBa_B0015, iGEM registry) using primers *B0015-fw* and *B0015-rev* and cloned between SacI and BamHI restriction sites of *pTn7-lux*, producing pTn*7-lux-B0015*. The *X. citri hrcU* promoter was PCR-amplified with primers *Pxac0406-F-PstI* and *Pxac0406-R-hindIII*, digested with PstI and HindIII and ligated into pTn7-lux and *pTn7-lux-B0015*, generating PhrcU and PhrcU-15, respectively. All primers used are listed in Table 1.

**Table 1.** Primers used in the study

Primer name	Sequence (5′-3′)
*pGLR-lux-fwd*	GCGATGGCCCTGTCCTTTTACC
*pGLR-lux-kpni-rev*	ATGCGGTACCGGGGACCCCTGGATTCTCACC
*B0015-fw-sacI*	AGTCGAGCTCCGGCCGCTTCTAGAGCCAG
*B0015-rev-ncoI-salI*	GATCGGATCCCCATGGAAGGGCAGGGTGGTGACAC
*Pxac0406-F-PstI*	ATACTGCAGGCAGGCAGCAGGCGTACAA
*Pxac0406-R-hindIII*	ATAAAGCTTTCCGACATTGCCCTCTCC
*glmS1-up*	AGCACCGCCTGCTGGAGA
*glmS1-down*	GATGTCGGCGACCGCTTTG
*Tn7L*	ATTTGCTTACGACGCTACACC
*Tn7R2*	AGGAGTCCAAGCTCAGCTAAT
*Tn7-seq2-fw*	TGTCAATTCGATCCATTGCTGT
*Tn7-seq2-rev*	TGGCAGGTAAACACTATTATCACCA

### Chromosomal insertions

Chromosomal integration was performed using the Tn7 transposition system [8]. Briefly, to prepare electrocompetent cells, * X. citri* cultures were grown in LB (Lysogeny Broth) to late exponential phase (OD ~1), washed three times with sterile deionized H₂O and then concentrated 100-fold. For each electroporation, 50 µl of competent cells was mixed with 100 ng each of the helper plasmid pTNS3 [9] and the Tn7 donor vector and electroporated at 20 kV cm^−1^. After recovering for 4 h in LB at 28 °C, cells were plated on LB agar supplemented with gentamicin (5 µg ml^−1^) and ampicillin (100 µg ml^−1^). Colony PCR was performed to confirm site-specific insertion using the primer set *glmS1-up/glmS-down* and the transposon-specific primers *Tn7L*, *Tn7R2*, *Tn7-seq2-fw* and *Tn7-seq2-rev* (Table 1). All chromosomal insertions were performed in *X. citri* pv. *citri* strain 306.

### Growth conditions and luminescence measurements

For single-point experiments, cultures grown overnight in LB were washed and diluted to OD_600nm_ 0.01 in test tubes containing 2 ml of either XVM2 medium [20 mM NaCl, 10 mM (NH_4_)_2_SO_4_, 5 mM MgSO_4_, 1 mM CaCl_2_, 0.16 mM KH_2_PO_4_, 0.32 mM K_2_HPO_4_, 0.01 mM FeSO_4_, 10 mM fructose, 10 mM sucrose, 0.03% Casamino acids (pH 6.7)] or XVM2-glucose [sucrose and fructose in XVM2 were replaced by glucose 0.5% (w/v)] and were grown for 20 h at 28 °C, with shaking. For carbon-switch experiments, cells were first grown in XVM2-glucose to mid-exponential phase (OD_600nm_ ~ 0.45), washed twice in carbon-free XVM2-C medium and then resuspended in either XVM2 or XVM2 containing 0.5% (m/v) of either galactose, glucose or xylose. Pairwise comparisons at the 8 h timepoint were performed using Student’s t-test with Bonferroni correction for multiple comparisons. Effect sizes are reported as fold change relative to glucose, with 95% CIs calculated on log-transformed data. Cultures were dispensed into sterile 96-well plates (200 µl per well) at 28 °C, with shaking. Luminescence and OD readings were measured every hour for 8 h. For both single-point and kinetics experiments, luminescence and OD_600_ were measured using a Varioskan^™^ LUX Multimode Microplate reader (Thermo Fisher Scientific, USA). Normalized luminescence values were calculated as luminescence divided by OD.

## Results and discussion

**Fig. 2.**
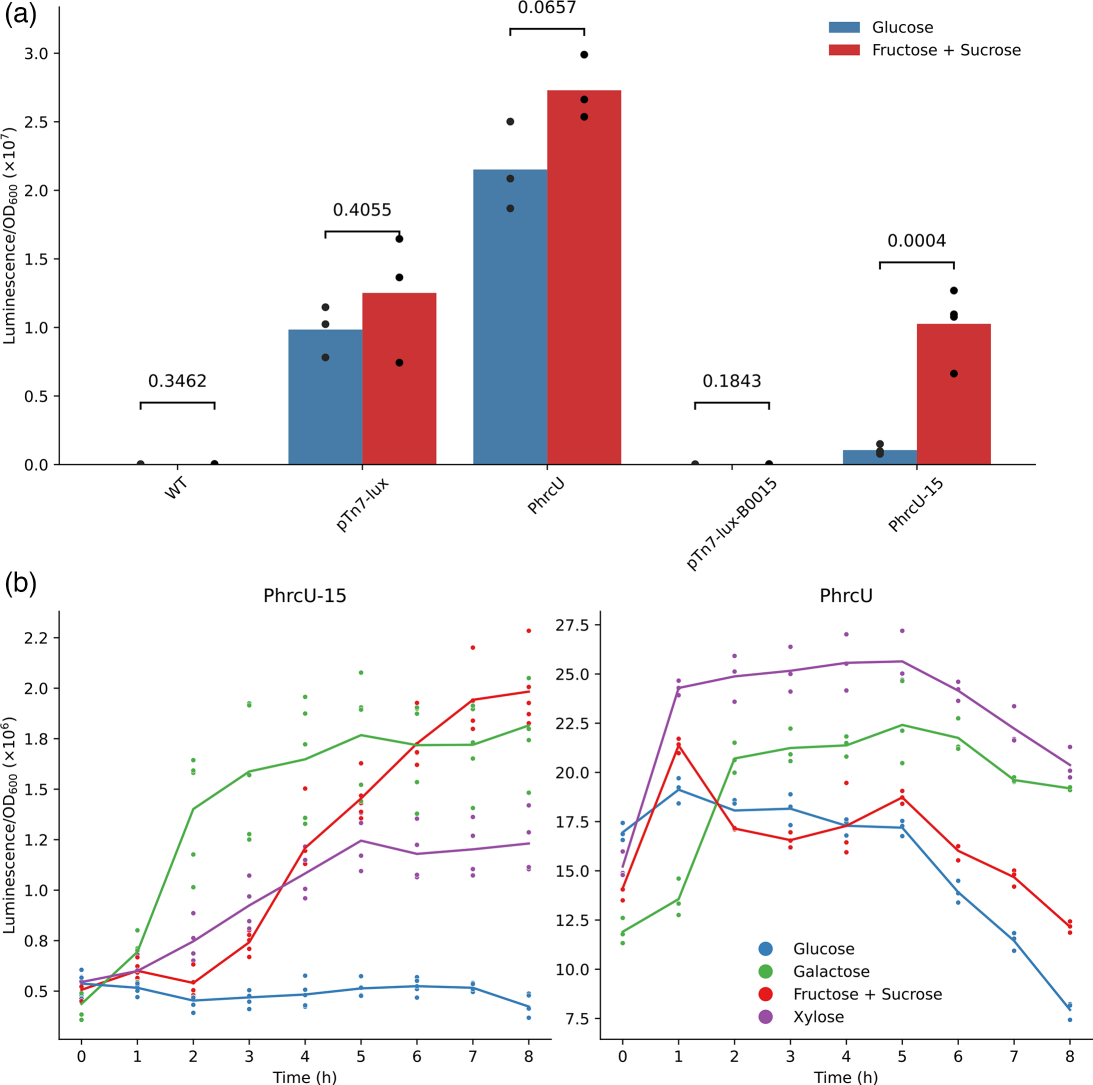
Characterization of T3SS gene expression sensors containing transcriptional fusions of the *hrcU* gene promoter (P*_hrcU_*) to the *lux* operon. (**a**) Normalized luminescence of *X. citri* strains with chromosomal insertions of P*_hrcU_* fusions (PhrcU and PhrcU-15) and empty vectors (pTn7-lux and pTn7-lux-B0015). Luminescence was measured from mid-exponential phase cultures grown either in XVM2 or XVM2-glucose. Numbers above the bars represent two-tailed *P* values calculated using an unpaired Student’s t-test. Bars represent the mean of at least three biological replicates, and points represent each biological replicate. (**b**) Promoter activity of the different constructs in response to carbon source switch. Bacteria were cultivated in XVM2-glucose until mid-exponential phase, washed twice in XVM2-C and resuspended in XVM2 containing the indicated sugars as the main carbon sources. Results are described as normalized luminescence. Solid lines represent the average of at least three biological replicates, and points represent each biological replicate.

Previously reported mini-Tn7-based plasmids suitable to generate promoter fusions to luminescent reporters have relatively large uncharacterized sequences in their MCSs (Fig. 1b). Therefore, we first sought to build a vector with minimal uncharacterized sequences between the MCS and the *lux* operon. For that, we created the pTn7-lux plasmid (Fig. 1a) by cloning the *luxCDABE* operon from the pGLR1 plasmid [7] into the pUC18T-mini-Tn7T-Gm plasmid, generating a vector with 94 bp of intervening sequence between the FRT site and the reporter gene. Because our group is interested in the regulation of the *X. citri* T3SS gene expression, we sought to create genetic sensors to follow the expression of virulence-related genes. For that, we cloned the promoter region of the gene *hrcU*, a T3SS structural gene from *X. citri*, into pTn7-lux and inserted it into X. citri’s chromosome (the construct hereafter called PhrcU). We then cultivated *X. citri*::PhrcU until mid-exponential phase in XVM2 synthetic medium [5], which has fructose and sucrose as carbon sources, or XVM2-glucose, conditions known for high and low expression of * X. citri*’s T3SS genes, respectively [6]. Surprisingly, both a promoterless insertion and the PhrcU fusion showed high normalized luminescence values in both conditions, and the expected increase in promoter activity by growth in XVM2 containing sucrose and fructose was not observed (Fig. 2a). This result suggested that another region with an unknown promoter sequence is driving the expression of the reporter genes.

In a previous report, removal of the antibiotic resistance cassette resulted in a 96% reduction in basal *luxCDABE* expression [4]. This suggests that expression may be driven by an upstream element located within the region flanked by the FRT sites. To address this, one approach is to excise the resistance marker using Flp recombinase. While marker removal is essential for developing strains suitable for industrial and environmental applications, retaining the antibiotic resistance gene can be advantageous in certain contexts, such as competition assays or recovering strains from complex experimental settings. To circumvent the requirement for antibiotic resistance marker removal and to reduce potential effects on the reporter expression, we inserted the double transcriptional terminator B0015, composed of the *E. coli rrnB* and T7 terminators, between the FRT and MCS regions of pTn7-lux, creating the pTn7-lux-B0015 plasmid (Fig. 1a).

To validate the redesigned vector, we integrated pTn7-lux-B0015 into *X. citri*’s chromosome and measured luminescence as above. We did not observe any significant difference in normalized luminescence compared to a wild-type strain (i.e. without any chromosomal insertion), confirming that this simple modification abolished any upstream promoter activity that may influence the expression of the genes of interest. To evaluate whether the double terminator could enhance the performance of a genetic sensor, we cloned the PhrcU promoter into pTn7-lux-B0015 to generate PhrcU-15, integrated it into the *X. citri* chromosome and tested it as before. In XVM2 medium, PhrcU-15 exhibited a ninefold increase in normalized luminescence compared to growth in XVM2-glucose (Fig. 2a). These results are consistent with previous studies that showed induction of *hrcU* gene expression in this medium, indicating that eliminating non-specific promoter activity from the original reporter vector is necessary for a reliable assessment of the promoter of interest [10].

To further analyse the responsiveness of the different promoter fusions to changes in environmental cues, we performed a carbon-switch experiment. Bacteria were first grown in XVM2-glucose until mid-exponential phase, then washed in carbon-free XVM2-C medium and resuspended in either XVM2, XVM2-glucose, XVM2-xylose or XVM2-galactose. Notably, we observed activation of PhrcU-15 in the presence of xylose and galactose, with the latter showing a faster activation compared to sucrose and fructose, although it reached a plateau within 3 h (Fig. 2b). At 8 h, PhrcU-15 showed significantly higher normalized luminescence in all carbon sources when compared to glucose: fructose (4.7-fold; 95% CI: 4.0–5.6; *P*<0.001), galactose (4.3-fold; 95% CI: 3.5–5.2; *P*<0.001) and xylose (2.9-fold; 95% CI: 2.4–3.6; *P*<0.001) (Fig. 2b). In contrast, PhrcU showed a high basal normalized luminescence, which decreased during the 8 h incubation in XVM2 and XVM2-glucose (Fig. 2b). On the other hand, the change to xylose and galactose led to a small increase in normalized luminescence relative to glucose (galactose: 2.4-fold, 95% CI: 2.1–2.8, *P*<0.001; xylose: 2.6-fold, 95% CI: 2.3–2.9, *P*<0.001), although raw luminescence increased less than twofold (1.4 and 1.7 for galactose and xylose, respectively; data not shown). Therefore, luminescence patterns suggest that the differences observed for PhrcU arise from differential maintenance or decay of background expression rather than authentic promoter activation. Thus, the terminator-containing construct not only improves endpoint fold change but also provides clearer and earlier kinetic resolution of promoter induction.

These results highlight the importance of eliminating cryptic regulatory elements when designing single-copy genetic reporters for quantitative gene expression studies. Our findings demonstrate that the widely used pUC18-mini-Tn7T-Gm-lux vector and its derivatives can exhibit substantial reporter activity in the absence of an inserted promoter, likely due to sequences near the antibiotic resistance cassette. By introducing a strong synthetic terminator upstream of the MCS, we successfully abolished background expression, enabling reliable detection of promoter-driven bioluminescence in response to environmental cues. The improved construct, pTn7-lux-B0015, offers enhanced dynamic range and temporal resolution, as demonstrated by the rapid and robust response to carbon source switching. This redesigned vector is a valuable tool for engineering sensitive and specific bioluminescent genetic sensors, particularly in applications where antibiotic resistance marker removal is not feasible or desired.

## Abbreviations

FRT: flippase recombinase target
MCS: multiple cloning site
T3SS: type III secretion system
